# Prelacteal feeding among infants within the first week of birth in eastern Uganda: evidence from a health facility-based cross-sectional study

**DOI:** 10.1186/s13006-021-00425-w

**Published:** 2021-10-12

**Authors:** Racheal Akello, Derrick Kimuli, Stephen Okoboi, Alimah Komuhangi, Jonathan Izudi

**Affiliations:** 1 Institute of Public Health, Clarke International University, P.O. Box 7782, Kampala, Uganda; 2 Directorate of Socio-Economic Surveys, Uganda Bureau of Statistics, P.O. Box 7186, Kampala, Uganda; 3 Infectious Diseases Institute, Makerere University College of Health Sciences, P.O. Box 22418, Kampala, Uganda; 4 Department of Community Health, Faculty of Medicine, Mbarara University of Science and Technology, P.O. Box 1410, Mbarara, Uganda

**Keywords:** Prelacteal feeding, Early breastfeeding initiation, Exclusive breastfeeding, Uganda

## Abstract

**Background:**

Prelacteal feeding hinders early initiation of breastfeeding and exclusive breastfeeding but is understudied in Uganda. We examined the prevalence and factors associated with prelacteal feeding among postpartum mothers in Kamuli district in rural eastern Uganda.

**Methods:**

We conducted a cross-sectional study between December 2020 and January 2021 at four large healthcare facilities and randomly sampled mother-baby pairs attending postnatal care and immunization clinics. Prelacteal feeding was defined as giving anything to eat or drink to a newborn other than breast milk within the first 0–3 days of life. Data were collected using a researcher-administered questionnaire and summarized using frequencies and percentages. The Chi-squared, Fisher’s exact, and Student’s t-tests were used for comparison while the factors independently associated with prelacteal feeding were determined using modified Poisson regression analysis, reported as an adjusted prevalence risk ratio (aPRR) with corresponding 95% confidence intervals (CI).

**Results:**

Of 875 participants enrolled, 319 (36.5%) practiced prelacteal feeding. The likelihood of prelacteal feeding was lower among participants who were unemployed (aPRR 0.70; 95% CI 0.5, 0.91), married (aPRR 0.71; 95% CI 0.58, 0.87), had received health education on infant feeding practices (aPRR 0.72; 95% CI 0.60, 0.86), had a spontaneous vaginal delivery (aPRR 0.76; 95% CI 0.61, 0.95), had delivered in a health facility (aPRR 0.73; 95% CI 0.60, 0.89), and who knew that prelacteal feeding could lead to difficulties in breathing (aPRR 0.70; 95% CI 0.57, 0.86). Conversely, prelacteal feeding was more likely among participants who had attended antenatal care at a public health facility during the most recent pregnancy (aPRR 2.41; 95% CI 1.71, 3.39) and those who had travelled more than 5 km to a health facility for postnatal care services (aPRR 1.46; 95% CI 1.23, 1.72).

**Conclusions:**

The prevalence of prelacteal feeding among postpartum mothers in rural eastern Uganda is slightly higher than the national average. Accordingly, there is a need to continuously educate mothers and staff on infant feeding practices to tackle the factors influencing prelacteal feeding and promote appropriate infant and young child feeding practices as emphasized in the baby-friendly health facility initiative policy.

## Background

Although breastfeeding is a common practice in most societies, prelacteal feeding remains a barrier to its promotion. A multi-level analysis of data from 22 countries in sub-Saharan Africa for risk factors of prelacteal feeding found a 32.2% prevalence of prelacteal feeding, with the likelihood being higher among mothers with a low level of education, preceding birth intervals of less than 24 months, low antenatal care attendance, home delivery, and small-sized baby at birth [1]. In East Africa, the pooled prevalence of prelacteal feeding is 12% [2]. One systematic review and meta-analysis conducted in Ethiopia reports a 25.3% pooled prevalence for prelacteal feeding, with the likelihood being lower among mothers that attended antenatal care during the most recent pregnancy, received counseling on infant feeding practices, timely initiated breastfeeding, and resided in an urban setting. However, prelacteal feeding is reported to be more likely among mothers who had given birth at home [3]. Other studies report that not being aware of the risks associated with prelacteal feeding and late initiation of breastfeeding [4], attending less than four antenatal care visits [5], maternal illiteracy and lack of breastfeeding counseling [6], misconceptions about breastfeeding [7], and cesarean section delivery [8] among others, are associated with increased likelihood of prelacteal feeding.

The 2016 Uganda Demographic and Health Survey data show that 66% of infants receive exclusive breastfeeding [9], thus feeding the infants only on breastmilk within the first 6 months of life. The report further showed that 34% of postpartum mothers practice prelacteal feeding, with 7% of newborn babies reported having received plain water, 6% non-milk liquids, 8% other milk, 11% complementary foods to breastmilk, and 2% no breastmilk at all [9]. Prelacteal feeding is therefore a common practice in Uganda but is understudied. Moreover, prelacteal feeding negatively impacts nutrition and breastfeeding compliance.

It is a major predisposing factor to thousands of infant deaths in developing countries and accounts for a large proportion of diarrheal and acute respiratory infections [10]. Giving newborn babies prelacteal feeds before colostrum exposes them to allergies and affects stimulation of breast milk production, suckling, and bonding with the mother.

To the best of our knowledge, only one study has been conducted on prelacteal feeding in eastern Uganda and the study found that more than half of postpartum mothers practiced prelacteal feeding in the first 3 days of newborn life [11]. Another study conducted in Western Uganda found that slightly more than three in every 10 postpartum mothers engage in prelacteal feeding [12]. However, the evidence presented in the study in Eastern Uganda is dated more than 10 years ago while the one in Western Uganda is nearly 10 years ago. Therefore, the evidence from the previous studies is obsolete and might not be appropriate to provide credible information on the current status of prelacteal feeding in eastern region. Recent data are therefore needed to understand prelacteal feeding in Uganda. In Kamuli district in Eastern Uganda, unpublished program data show that postpartum mothers practice prelacteal feeding. However, data describing the magnitude of prelacteal feeding and the associated factors are lacking. We examined the prevalence and factors associated with prelacteal feeding in the district. This information will help in designing context-specific interventions to tackle prelacteal feeding in the district and the rest of the districts in Eastern Uganda, including similar regions in Uganda and sub-Saharan Africa.

## Methods

### Study design and setting

This was a health facility-based cross-sectional study conducted between December 2020 and January 2021 at four health facilities with large patient numbers in Kamuli District. We preferred to conduct a health facility-based study since the majority of mothers in the community come to these study sites for delivery and immunization services, and because it was not logistically efficient to conduct a community-based study. Besides serving the majority of the population in the district, the health facilities are sites for most postnatal care visits. The district has a substantially higher number of newborn deaths which has been linked to prelacteal feeding [13]. For example, a review of data from the District Health Information System-2 or DHIS-2 for 3 years showed higher neonatal mortality rates of 6.57 deaths per 1000 live births compared to 6.5 deaths per 1000 live births at the national level in 2017/2018. For 2018/2019, there were 7.2 deaths per 1000 live births in the district versus 7.7 deaths per 1000 live births at the national level, while for 2019/2020, the district had 6.2 deaths per 1000 live births compared to 7.1 deaths per 1000 live births at the national level. The district also faces nutritional challenges [14]. Data further suggest that 37.1% of children below the age of five in the district are stunted, which is far beyond the national stunting rate of 29.0% [9]. Additionally, 22.7% of the children are underweight which is higher than the national average of 11.0%, and an estimated 16.7% of children are wasted which is more than 4-fold the national average at 4% [9]. With merely 36.6% of health facilities in the district with the capacity to manage childhood malnutrition, these nutritional health problems will continue to present real public health challenges [13].

The study sites included three public health facilities namely Kamuli General Hospital, Namwendwa Health Center IV, Nankandulo Health Center IV, and one private-not-for-profit health facility, Kamuli Mission Hospital. Kamuli District is located in East Central Uganda and has an estimated population size of 545,900 people [15]. Each health facility has a maternal and child health (MCH) clinic which offers antenatal care, delivery, and postnatal care services. Antenatal care services are provided daily to ensure service continuity. Mothers are encouraged to attend up to eight antenatal care visits throughout their pregnancy. At each visit, various services are provided including maternal-child health education and individual counseling. At each antenatal and postnatal care visit, women receive education and counseling about maternal nutrition, are assessed for nutritional status using mid-upper arm circumference and weights, and receive information about healthy breastfeeding and infant feeding practices. Iron and folic acid supplementation and deworming are prescribed per the national ANC guideline. After delivery, mothers are encouraged to initiate breastfeeding within the first hour of birth. Delivery services are provided 24 h a day, 7 days a week by midwives and/or doctors. Postnatal care services are provided at 6 h, 24 h, 6 days, 6 weeks, and 6 months after delivery.

#### Implementation of baby-friendly health facility initiative at the study sites

The study sites implement the baby-friendly health facility initiative (BFHI), a 10 step intervention initiated by the World Health Organization (WHO) and the United Nations Children’s Emergency Fund (UNICEF) in 1991, which aims to promote, protect and support breastfeeding. The BFHI framework helps health facilities address breastfeeding practices that harm newborn babies [16]. BFHI largely emphasize the need for all pregnant women to receive information about the benefits and management of breastfeeding, supporting mothers with the initiation of breastfeeding within an hour of birth, and not giving food or drink other than breast milk to newborn babies unless medically indicated.

If well implemented, the BFHI framework is anticipated to tackle some of the factors which promote prelacteal feeding and thus contribute to promoting appropriate infant and young child feeding practices among breastfeeding mothers. Uganda adopted the BFHI framework but added six more steps to ensure mothers are supported to acquire skills to exclusively breastfeed for 6 months. Health facilities are assessed and supported to achieve these 16 steps through internal and external assessment and support mechanisms. However, not much progress has been registered on the implementation of the BFHI steps, especially steps focusing on prelacteal feeding practices due to limited funding and knowledge inadequacy among health workers [17].

### Study population and sampling

The study population consisted of mother-baby pairs aged 4–42 days attending postnatal care and immunization clinics at the respective study sites. We excluded newborn babies whose biological mothers had died because we deemed that the practice of prelacteal feeding would be almost inevitable. Since prelacteal feeding occurs within 0–3 days, we excluded mother-baby pairs within this period. The mother-baby pairs were sampled via systematic and simple random sampling approaches. First, we proportionally allocated the required sample size to each of the four study sites based on the number of deliveries. We then employed systematic random sampling to establish the sampling interval at each of the study sites. To do so, we reviewed records to establish the number of mother-baby pairs present at the postnatal and immunization clinics on a particular day and this formed our sampling frame. We assigned unique codes to each mother-baby pair in the sampling frame. We divided the number of postpartum mother-baby pairs at each clinic by the site’s sample size to obtain the sampling interval. We then used a simple random sampling approach, a lottery method, with a random start to select the first and subsequent participants until all the required number of participants was reached.

### Study variables and measurements

The dependent variable was prelacteal feeding measured as giving anything to eat or drink to a newborn baby other than breast milk within the first 0–3 days of life, a definition based on the Uganda Demographic Health Survey [9] and previous literature [3, 6, 18]. The independent variables included maternal age, ethnicity, level of education, type of employment, marital status, religion, and HIV status established from health facility records, and the number of antenatal care visits at the recent pregnancy. Others included birth order, place and mode of delivery, maternal residence, and knowledge about the risks of prelacteal feeding. We also collected data on the level of health facility, place of recent antenatal care attendance, and the estimated distance from the place of residence to the health facility.

### Data collection and processing

Data were collected within the health facility premises in a quiet and convenient room using a pre-tested researcher-administered questionnaire in the local language, *Lusoga.* On average, the administration of the questionnaire lasted 30–45 min. Each filled questionnaire was checked for completeness in real-time before the data were entered in Epi-Data version 3.1. We employed data quality control measures impregnated in Epi-Data such as range and legal values, skips, and alerts to ensure data integrity.

### Sample size estimation and statistical analysis

Two approaches were used to establish the required sample size. Based on the prevalence of prelacteal feeding in a previous Ugandan study, a sample size of 377 participants was required using Kish and Leslie formula when the following assumptions were made: 57% prevalence of prelacteal feeding among children aged 6–24 months [19], 95% confidence level, and 5% maximum allowable error. To determine factors associated with prelacteal feeding, the sample size was estimated using the two proportions sample size estimation approach. Based on estimates from a previous cross-sectional study in South Sudan [20], half of the postpartum mothers who never received breastfeeding counseling engaged in prelacteal feeding while among those who received breastfeeding counseling, 60% had engaged in prelacteal feeding. We estimated that 875 participants would be needed to ensure 80% statistical power in detecting a true difference at a 95% confidence level. Accordingly, the study used the large sample size to minimize biased estimation of the measure of effect.

Concerning statistical analysis, in the univariate analysis, we computed frequencies and percentages for categorical data. For numerical data, we computed means with standard deviation when the data were not skewed, otherwise, the median with interquartile range was computed. In the bivariate analysis, we compared differences in prelacteal feeding with the categorical independent variables using the Chi-squared test for larger cell counts, otherwise, Fisher’s exact test was employed for smaller cell counts. Mean differences in prelacteal feeding with numerical independent variables were established using the Student’s t-test when the data were normally distributed, otherwise the Wilcoxon-rank sum test was used. The level of statistical significance was set at less than 0.15 to avoid residual confounding.

In the multivariate analysis, we computed both unadjusted (crude) and adjusted prevalence risk ratios with the corresponding 95% confidence intervals using modified Poisson regression analysis with robust error variance for all statistically significant variables at the bivariate analysis. The prevalence risk ratio (PRR) was preferred over the odds (OR) to minimize overestimation since the outcome of interest, prelacteal feeding, was large [21]. Robust error variance was used to ensure convergence and avoid mild violations of the assumptions of Poisson regression as recommended by Trivedi and Cameron [22]. Variables with *p* < 0.05 were considered statistically significant. We assessed the model fitness using Akaike Information Criteria (AIC), Hosmer-Lemeshow Chi-square goodness-of-fit statistics, and link test. In the multivariate analysis, we dropped variables that did not improve the fit of the model based on the log-likelihood. The analysis was conducted in Stata version 15.

### Ethical issues

Our study was reviewed and approved by Clarke International University Research Ethics Committee (reference # CLARKE-2020-23). Administrative approval was obtained from the District Health Office, Kamuli district, and the Heads of the respective study sites. All the participants were informed about the purpose of the study, confidentiality of information, privacy, the benefits and potential risks involved in the study, and the potential to withdraw at any time. The participants provided written or thumb-printed informed consent before participation. Access to data was restricted to the study team and data were safely secure in a password-protected computer accessible by only the data analyst. Besides the use of unique codes on the questionnaire, data about personal identifiers such as names and physical addresses were not collected.

## Results

### General characteristics of the participants

Table 1 shows the general characteristics of the study participants. A total of 875 participants with a mean age of 26.2 ± 5.9 were enrolled in the study. Of all the participants, 491 (56.1%) were aged 25 years or older, 611 (69.8%) were of the Basoga ethnicity, 388 (44.3%) had secondary education as their highest level of education, 437 (49.9%) had no employment, 710 (81.1%) were married, 637 (75.4%) were Catholic, and 80 (9.1%) were mothers living with HIV.

**Table 1:** General characteristics of the participants

Characteristics	Levels	Total (***n*** = 875)
Age categories in years	15–24	384 (43.9)
	25 and beyond	491 (56.1)
	mean (SD)	26.2 (5.9)
Ethnicity	Basoga	611 (69.8)
	Baganda	103 (11.8)
	Basamia	23 (2.6)
	Mugishu	21 (2.4)
	Others	117 (13.4)
Level of education	None	83 (9.5)
	Primary	323 (36.9)
	Secondary	388 (44.3)
	Tertiary and beyond	81 (9.3)
Type of employment	Formal	102 (11.7)
	Self	336 (38.4)
	None	437 (49.9)
Marital status	Single/never married	116 (13.3)
	Married	710 (81.1)
	Divorced/separated	49 (5.6)
Religion	Catholic	637 (75.4)
	Muslim	195 (23.1)
	Others	13 (1.5)
A mother living with HIV	No	795 (90.9)
	Yes	80 (9.1)
Number of antenatal care visits at recent pregnancy	Less than 4	651 (74.4)
	4 and more	224 (25.6)
	mean (SD)	3.7 (1.7)
Birth order	First	181 (20.7)
	Second	227 (25.9)
	Third	197 (22.5)
	Fourth	112 (12.8)
	Fifth and beyond	158 (18.1)
Place of delivery	Outside a Health facility	148 (16.9)
	In a Health facility	727 (83.1)
Maternal residence	Urban	194 (22.2)
	Peri-urban	335 (38.3)
	Rural	346 (39.5)
Level of health facility	Health center	368 (42.1)
	General Hospital	507 (57.9)
Place of recent antenatal care attendance	Private-not-for profit	200 (22.9)
	Public/or government	675 (77.1)
Distance from home to health facility (km)	Less or equals 5 km	510 (58.3)
	Beyond 5 km	365 (41.7)
	Mean (SD)	7.0 (8.2)

The majority of the participants had attended less than four antenatal care visits at the most recent pregnancy (651 or 74.4%), 227 (25.9%) had a baby with a second birth order, 727 (83.1%) had delivered in a health facility, and 346 (39.4%) lived in a rural area. Furthermore, the majority of the participants attended postnatal care at a general hospital (507 or 57.9%) and public health facility (675 or 77.1%). 510 (58.3%) travelled ≤5 km from their place of residence to the health facility for postnatal care services. The mean distance travelled was 7.0 ± 8.2 km.

### Prelacteal feeding and the relationship with personal and health services related factors

Table 2 summarizes the results for the comparison of differences in prelacteal feeding with personal and health service-related factors. Our data show that 319 (36.5%) participants practiced prelacteal feeding. Participants who practiced prelacteal feeding were on average similar to those who never practiced prelacteal feeding: 26.4 ± 6.2 versus 26.1 ± 5.7 years, *p* = 0.491. Prelacteal feeding was more common among participants aged 25 years and beyond (37.1%), other ethnic tribe (45.3%), those without any formal education (44.6%), the formally employed (43.1%), the single or never married (51.7%), and those living with HIV (40.1%).

**Table 2:** Prevalence of prelacteal feeding and the relationship with personal and health services related factors

Characteristics	Levels	Prelacteal feeding		
		No (***n*** = 556)	Yes (***n*** = 319)	***P*** - value
Age categories	15–24	247 (64.3)	137 (35.7)	0.672
	25 and beyond	309 (62.9)	182 (37.1)	
	mean (SD)	26.1 (5.7)	26.4 (6.2)	0.491
Ethnicity	Basoga	404 (66.1)	207 (33.9)	0.058
	Baganda	58 (56.3)	45 (43.7)	
	Basamia	17 (73.9)	6 (26.1)	
	Mugishu	13 (61.9)	8 (38.1)	
	Others	64 (54.7)	53 (45.3)	
Level of education	None	46 (55.4)	37 (44.6)	0.375
	Primary	212 (65.6)	111 (34.4)	
	Secondary	248 (63.9)	140 (36.1)	
	Tertiary and beyond	50 (61.7)	31 (38.3)	
Type of employment	Formal	58 (56.9)	44 (43.1)	0.003
	Self	196 (58.3)	140 (41.7)	
	None	302 (69.1)	135 (30.9)	
Marital status	Single/never married	56 (48.3)	60 (51.7)	< 0.001
	Married	473 (66.6)	237 (33.4)	
	Divorced/separated	27 (55.1)	22 (44.9)	
A mother living with HIV	No	508 (63.9)	287 (36.1)	0.490
	Yes	48 (60.0)	32 (40.0)	
Number of antenatal care visits at recent pregnancy	Less than 4	396 (60.8)	255 (39.2)	< 0.001
	4 and more	160 (71.4)	64 (28.6)	
	mean (SD)	6.06 ± 1.91	5.24 ± 2.52	< 0.0001
Birth order	First	124 (68.5)	57 (31.5)	0.158
	Second	151 (66.5)	76 (33.5)	
	Third	122 (61.9)	75 (38.1)	
	Fourth	70 (62.5)	42 (37.5)	
	Fifth and beyond	89 (56.3)	69 (43.7)	
Mode of delivery	Caesarean section	69 (54.3)	58 (45.7)	0.020
	Spontaneous vaginal delivery	487 (65.1)	261 (34.9)	
Delivered in a health facility	No	63 (42.6)	85 (57.4)	< 0.001
	Yes	493 (67.8)	234 (32.2)	
Birthweight (kg)	Less than 2.5	20 (47.6)	22 (52.4)	0.089
	2.5–4.0	478 (64.3)	265 (35.7)	
	Above 4.0	58 (64.4)	32 (35.6)	
Maternal residence	Urban	121 (62.4)	73 (37.6)	0.828
	Peri-urban	217 (64.8)	118 (35.2)	
	Rural	218 (63.0)	128 (37.0)	
Prelacteal feeding causes diarrhea	No	134 (60.6)	87 (39.4)	0.299
	Yes	422 (64.5)	232 (35.5)	
Prelacteal feeding causes breathing difficulties	No	362 (59.2)	249 (40.8)	< 0.001
	Yes	194 (73.5)	70 (26.5)	
Level of health facility	Health center	220 (59.8)	148 (40.2)	0.049
	General Hospital	336 (66.3)	171 (33.7)	
Place of recent antenatal care attendance	Private-not-for profit	170 (85.0)	30 (15.0)	< 0.001
	Public/or government	386 (57.2)	289 (42.8)	
Distance from home to health facility (km)	Less or equals 5 km	354 (69.4)	156 (30.6)	< 0.001
	Beyond 5 km	202 (55.3)	163 (44.7)	
	Mean (SD)	5.7 (6.5)	9.3 (10.1)	< 0.0001

*Note***:** 1) Number in parenthesis (brackets) are row percentages calculated as n/N, where n is the frequency and N is the total number of participants in the row. 2) SD denotes standard deviation

Participants who attended less than four antenatal care visits at the most recent pregnancy (39.2%), gave birth to the fifth child or more (43.9%), did not deliver in a health facility (57.4%), and resided in an urban setting (37.6%) had a higher prevalence of prelacteal feeding. The distribution of prelacteal feeding by knowledge of risks of diarrhoea and breathing difficulties, place of antenatal and postnatal care attendances, and travel distance is equally shown in Table 2.

We observed statistically significant differences in prelacteal feeding concerning the type of employment (*p* = 0.003), marital status (*p* < 0.001), number of antenatal care visits at the most recent delivery (*p* < 0.001), mode of delivery (*p* = 0.020), place of delivery (*p* < 0.001), knowledge of whether prelacteal feeding causes breathing difficulties or not (*p* < 0.001), level of health facility (*p* = 0.049), place of recent attendance of antenatal care (*p* < 0.001), and distance travelled from place of residence to a health facility for postnatal care (*p* < 0.001).

### Factors associated with prelacteal feeding at unadjusted and adjusted analysis

In the unadjusted analysis (Table 3), prelacteal feeding was less likely when the participant was unemployed (PRR 0.72; 95% CI 0.55, 0.93), married (PRR 0.65; 95% CI 0.53, 0.79), had attended four or more antenatal care visits at the most recent pregnancy (PRR 0.73; 95% CI 0.58, 0.92), had received health education on infant feeding practices during antenatal care visits (PRR 0.53; 95% CI 0.45,0.63), had a spontaneous vaginal delivery (PRR 0.56; 95% CI 0.47,0.67), had delivered in a health facility (PRR 0.56; 95% CI 0.47,0.67), had given birth to a newborn that had a birthweight of 2.5–5.0 kg (PRR 0.68; 95% CI 0.50, 0.92), knew that prelacteal feeding could cause breathing difficulties (PRR 0.65; 95% CI 0.52, 0.81), had given the baby colostrum (PRR 0.50; 95% CI 0.40, 0.61), and had attended postnatal care at a general hospital (PRR 0.84; 95% CI 0.70, 0.99).

**Table 3:** Factors associated with prelacteal feeding at unadjusted and adjusted modified Poisson regression analysis

Characteristics	Level	Modified Poisson regression analysis	
		Unadjusted	Adjusted
		PRR (95% CI)	aPRR (95% CI)
Type of employment	Formal	1	1
	Self	0.97 (0.75,1.25)	0.89 (0.69, 1.15)
	None	0.72^*^(0.55,0.93)	0.70^**^ **(0.54, 0.91)**
Marital status	Single/never married	1	1
	Married	0.65^***^ (0.53,0.79)	0.71^***^(0.58, 0.87)
	Divorced/separated	0.87 (0.61,1.24)	0.89 (0.64,1.22)
Number of antenatal care visits at recent pregnancy	Less than 4	1	
	4 and more	0.73^**^ (0.58,0.92)	
Received health education on infant feeding at recent antenatal care visits	No	1	**1**
	Yes	0.53^***^ (0.45,0.63)	0.72^***^ **(0.60,0.86)**
Mode of delivery	Caesarean section	1	1
	Spontaneous vaginal delivery	0.76^*^(0.62,0.95)	0.76^*^ **(0.61,0.95)**
Delivered in a health facility	No	1	1
	Yes	0.56^***^ (0.47,0.67)	0.73^**^ **(0.60,0.89)**
Birthweight (kilograms)	Less than 2.5	1	
	2.5–4.0	0.68^*^(0.50,0.92)	
	Above 4.0	0.68 (0.45,1.01)	
Prelacteal feeding causes breathing difficulties	No	1	1
	Yes	0.65^***^ (0.52,0.81)	0.70^***^ **(0.57,0.86)**
Baby received colostrum	No	1	
	Yes	0.50^***^ (0.40,0.61)	
Level of health facility	Health Center	1	
	General Hospital	0.84^*^ (0.70,0.99)	
Place of recent antenatal care attendance	Private-not-for profit	1	1
	Public/or government	2.85^***^ (2.03,4.02)	2.41^***^ **(1.71,3.39)**
Distance from home to health facility (km)	Less or equals 5 km	1	1
	Beyond 5 km	1.46^***^ (1.23,1.74)	1.46^***^ **(1.23,1.72)**

*Note*: Exponentiated coefficients are for prevalence risk ratios; 95% confidence intervals in brackets; Significance codes at 5% level: ^*^ *p* < 0.05, ^**^ *p* < 0.01, ^***^ *p* < 0.001. PRR: Unadjusted prevalence risk ratio; aPRR: Adjusted prevalence risk ratio

However, antenatal care attendance at a public health facility during the most recent pregnancy (PRR 2.85; 95% CI 2.03, 4.02) and travel distance exceeding 5 km to access postnatal care services (PRR 1.46; 95% CI 1.23, 1.74) were associated with a higher likelihood of prelacteal feeding.

In the adjusted analysis (Table 3), the number of antenatal care visits at the most recent pregnancy, birth weight, receipt of colostrum, and the level of health facility did not improve the model fitness so they were dropped. Our final model was parsimoniously characterized by the following: the lowest Akaike Information Criteria (AIC) of 1207.2, a goodness-of-fit value of 533.9 (Chi-square = 864, *p* = 1.000), and a statistically insignificant *p* - value associated with a linktest (*p* = 0.807). Our data show that prelacteal feeding was less likely when the participant was unemployed (aPRR 0.70; 95% CI 0.50,0.91), married (aPRR 0.71; 95% CI 0.58, 0.87), had received health education on infant feeding at the most recent pregnancy (aPRR 0.7; 95% CI 0.60, 0.86), had a spontaneous vaginal delivery (aPRR 0.76; 95% CI 0.61, 0.95), had delivered in a health facility (aPRR 0.73; 95% CI 0.60, 0.89), and knew that prelacteal feeding could lead to difficulties in breathing (aPRR 0.70; 95% CI 0.57, 0.86). Conversely, prelacteal feeding was more likely when the participant had attended antenatal care at a public health facility during the most recent pregnancy (aPRR 2.41; 95% CI 1.71, 3.39) and when the participant had travelled more than 5 km to receive postnatal care services (aPRR 1.46; 95% CI 1.23, 1.72).

## Discussion

The focus of this study is on the prevalence and factors associated with prelacteal feeding in Kamuli district in rural Eastern Uganda. Our data show that at least three in every ten postpartum mothers practice prelacteal feeding, which is distant from the prevalence of prelacteal feeding reported in a previous study in South Sudan at 53% [20] and Eastern Uganda at 57% [11].

The variation could be due to differences in the study settings. The present study was conducted in a health facility setting while the previous studies were conducted in a community setting. Community-based studies often provide higher prevalence estimates compared to health facility-based studies due to systematic differences in the characteristics of the study participants [12]. Conversely, the present prevalence of prelacteal feeding is comparable with the findings of the 2016 Uganda Demographic and Survey which places the prevalence at 34% [9], and another health facility-based study in Western Uganda which reports a prevalence of 31.3% [12]. Therefore, our data show that the prevalence of prelacteal feeding is high and should be a concern for the healthcare system to address as it predisposes newborn babies to significant morbidity and mortality [10].

Our study shows that unemployed mothers are less likely to engage in prelacteal feeding compared to mothers with formal employment. Our finding is consistent with one Ethiopian study which found a higher likelihood of prelacteal feeding among mothers engaged in farming compared to housewives [23]. Our finding is also consistent with the findings of one study in Kenya which report breastfeeding challenges at the workplace where mothers had to resume work shortly after delivery and then work for longer hours, making them unable to breastfeed optimally [24]. The unemployed mothers in our study are mainly housewives, often without any form of employment in the formal or informal sectors. Our finding could be explained by differences in work demands between the unemployed and the employed mothers. For example, insufficient maternity leave days might have forced employed mothers to introduce prelacteal feeds to enable their early return to work. However, further studies are needed to explore this finding. Our finding highlights the need to promote baby-friendly workplaces to allow mothers to freely breastfeed while working.

We found that married mothers are less likely to practice prelacteal feeding compared to single or separated mothers. A previous study in Ethiopia showed that single or never-married mothers rarely use existing maternal child health services compared to the married mothers [25]. Accordingly, married mothers tended to have sufficient information about breastfeeding practices compared to single or never-married mothers hence their lower chances of prelacteal feeding. Another possible explanation could be that married mothers tend to receive support from their spouses like encouragement to breastfeed and use maternal and child health services hence the observed difference.

Our data show a lower likelihood of prelacteal feeding among mothers who received health education on infant feeding practices during antenatal care visits compared to those who never received such information. Health education is important in demystifying cultural and traditional beliefs against breastfeeding and empowering mothers with the correct information about breastfeeding. Our finding is consistent with existing literature. Previous studies report that lack of counseling on breastfeeding [6], lack of information about the risks of prelacteal feeding [6, 8], and inadequacies of knowledge on breastfeeding practices [6] are associated with a higher likelihood of prelacteal feeding. Other studies report that counseling on breastfeeding is associated with a reduction in prelacteal feeding [3, 20].

Our study shows that prelacteal feeding is less likely among mothers who had spontaneous vaginal delivery compared to those who had a cesarean section delivery. Our finding is consistent with the results of previous studies [8, 26, 27]. The plausible biological explanation is that as a baby breastfeeds, the nipple is stimulated, and this causes the release of oxytocin into the maternal bloodstream resulting in the contraction of the uterine muscles.

Uterine contraction is usually associated with pain which potentially is much more pronounced in mothers who delivered by cesarean section compared to those who delivered through spontaneous vaginal delivery. Furthermore, discomfort in breastfeeding is experienced much more among mothers with cesarean section delivery compared to those with spontaneous vaginal delivery. The tendency to avoid breastfeeding and opt for prelacteal feeding is therefore highly likely. Also, cesarean delivery is associated with delays in breastfeeding initiation as the mother recovers from the surgery during which time prelacteal feeds are often used as she awaits recovery.

The study found that prelacteal feeding is less likely among mothers who deliver in a health facility compared to those who deliver at home. Home delivery is associated with a higher likelihood of prelacteal feeding in several studies in sub-Saharan Africa [3, 5, 6, 8]. This could be because mothers who deliver at home miss skilled attendance at birth resulting in poor immediate newborn and postnatal care. Mothers who deliver at home are also easily influenced by traditional birth attendants to give prelacteal feeds instead of immediate initiation of breastfeeding. Conversely, mothers who deliver in a health facility receive immediate information on correct infant feeding practices and this reduces the likelihood of prelacteal feeding.

Our study shows that prelacteal feeding is less likely among mothers who knew that the practice could lead to difficulties in breathing among newborn babies. This finding is consistent with the results of a previous Ethiopian study [28]. Our findings highlight the significance of empowering mothers with correct and adequate information about infant feeding. The healthcare system should ensure that every mother receives information about the risks associated with prelacteal feeding to mitigate the practice.

This study shows that mothers who attended antenatal services at a public health facility during the recent pregnancy are more likely to give prelacteal feeds compared to those who attended antenatal care services at a private-not-for-profit health facility. This could be attributed to the high workload at public health facilities compared to private-not-for profit health facilities resulting in a lack of ample time to provide health education messages or even counsel pregnant mothers about appropriate infant feeding practices [29]. There is also the possibility that this finding might have resulted from differences in sample sizes between the public and private-not-for profit health facilities, with most of the data analyzed drawn from the former than the latter health facilities.

We found that mothers who travel more than 5 km to a health facility to receive postnatal care services are more likely to practice prelacteal feeding compared to those who travel 5 km or less. This finding is consistent with the requirements of the Uganda National Health Policy framework [30]. Accordingly, a population that lives within a radius of 5 km to a health facility has easy access to health services while those who live beyond 5 km have difficult access to health services [31]. Our finding is thus an implication of difficult access to existing maternal and child health services. Longer travel distances thus present a physical barrier to seeking essential maternal and child health services due to the associated direct and indirect costs. This finding is consistent with a previous study that reported longer travel distance limits access to health services [32].

### Study strengths and limitations

Our study has several strengths and limitations. To the best of our knowledge, this is the first study on prelacteal feeding in the study setting. The study has a large sample size and is adequately powered to detect a statistically significant difference. The study was conducted at health facilities with the highest patient loads in the district so the results are likely representative.

However, notable limitations of the study include the lack of qualitative data to explain some of the reasons for prelacteal feeding. There is also the possibility of recall bias especially among mothers who were nearly 42 days postpartum. Another limitation is that our findings demonstrate association but not causation. Also, our study focused on mothers who accessed postnatal care services so there is a possibility of selection bias especially if mothers who never attended postnatal care services are systematically different from those who attended postnatal care services concerning residence, economic status, employment, and religion among others. Also, findings from a health facility-based study at times provide different estimates from community-based studies due to systematic differences between the study participants and this raises external validity concerns. These limitations should be considered in the interpretation of the results.

## Conclusions

Our study shows that prelacteal feeding is highly prevalent in rural eastern Uganda. The study demonstrates a need to continuously educate postpartum mothers on infant feeding practices to tackle the factors influencing prelacteal feeding and promote appropriate infant and young child feeding practices as emphasized in the baby-friendly health facility initiative policy. We implore policymakers to use these findings to inform policy formulation. Nutritionists, nurses, midwives, health educators, and village-level health workers should use these findings to design context-relevant health education messages about breastfeeding practices.

## Data Availability

The datasets used and/or analyzed during the current study are available from the corresponding author on reasonable request.

## Abbreviations

AIC: Akaike Information Criteria
aPRR: Adjusted Prevalence Risk Ratio
HIV: Human Immunodeficiency Virus
uPRR: Unadjusted Prevalence Risk Ratio

## References

[1] Berde AS, Ozcebe H (2017). Risk factors for prelacteal feeding in sub-Saharan Africa: a multilevel analysis of population data from twenty-two countries. Public Health Nutr.

[2] Birhan TY, Birhan NA, Alene M (2021). Pooled prevalence and determinants of prelacteal feeding practice in eastern Africa evidence from demographic and health survey data: a multilevel study. Risk Manag Healthc Policy.

[3] Temesgen H, Negesse A, Woyraw W, Getaneh T, Yigizaw M (2018). Prelacteal feeding and associated factors in Ethiopia: systematic review and meta-analysis. Int Breastfeed J.

[4] Legesse M, Demena M, Mesfin F, Haile D (2014). Prelacteal feeding practices and associated factors among mothers of children aged less than 24 months in Raya kobo district, North Eastern Ethiopia: a cross-sectional study. Int Breastf J.

[5] Bayih WA, Mekonen DK, Kebede SD (2020). Prevalence and associated factors of prelacteal feeding among neonates admitted to neonatal intensive care units, north Central Ethiopia, 2019. BMC Public Health.

[6] Sorrie MB, Amaje E, Gebremeskel F (2020). Pre-lacteal feeding practices and associated factors among mothers of children aged less than 12 months in Jinka town, South Ethiopia, 2018/19. PLoS One.

[7] Chea N, Asefa A (2018). Prelacteal feeding and associated factors among newborns in rural Sidama, South Ethiopia: a community based cross-sectional survey. Int Breastfeed J.

[8] Wolde TF, Ayele AD, Takele WW (2019). Prelacteal feeding and associated factors among mothers having children less than 24 months of age, in Mettu district, Southwest Ethiopia: a community based cross-sectional study. BMC Res Notes.

[9] Uganda Bureau of Statistics (UBOS) and ICF (2018). Uganda Demographic and Health Survey 2016.

[10] Bekele Y, Mengistie B, Mesfine F (2014). Prelacteal feeding practice and associated factors among mothers attending immunization clinic in Harari region public health facilities, eastern Ethiopia. Open J Prev Med.

[11] Engebretsen IMS, Tylleskär T, Wamani H, Karamagi C, Tumwine JK (2008). Determinants of infant growth in eastern Uganda: a community-based cross-sectional study. BMC Public Health.

[12] Ogah A, Ajayi A, Akib S, Okolo S (2012). A cross-sectional study of pre-lacteal feeding practice among women attending Kampala International University teaching hospital maternal and child health clinic, Bushenyi, Western Uganda. Asian J Med Sci.

[13] Musasizi B, Kiracho EE, Kamukama S, Babughirana G (2018). Assessment of public health units’ capacity to manage under-five malnutrition: a case study of Kamuli district, Uganda. Int J Stud Nurs.

[14] Uganda’s electronic health information system (eHMIS): dhis2 [https://hmis.health.go.ug/dhis-web-commons/security/login.action]. Accessed 28 July 2021.

[15] Uganda Bureau of Statistics. The National population and housing census 2014 – main report, Kampala, Uganda. vol. 6. 2016. p. 12–20.

[16] Lillehoj CJ, Dobson BL (2012). Implementation of the baby-friendly hospital initiative steps in Iowa hospitals. J Obstet Gynecol Neonatal Nurs.

[17] Geoffrey B, Benon M, Barungi LM, Tumuhameho A, Florence T, Rwegyema T (2016). Transforming health facilities into mother-baby friendly centers: experience of world vision, east African maternal newborn and child health project in Kitgum District, Uganda, 2016. Open Access Lib J.

[18] Takele WW, Tariku A, Wagnew F, Ekubagewargies DT, Getinet W, Derseh L, Anlay DZ (2018). Magnitude of prelacteal feeding practice and its association with place of birth in Ethiopia: a systematic review and meta-analysis, 2017. Arch Public Health.

[19] Engebretsen IM, Wamani H, Karamagi C, Semiyaga N, Tumwine J, Tylleskär T (2007). Low adherence to exclusive breastfeeding in eastern Uganda: a community-based cross-sectional study comparing dietary recall since birth with 24-hour recall. BMC Pediatr.

[20] Tongun JB, Sebit MB, Ndeezi G, Mukunya D, Tylleskar T, Tumwine JK (2018). Prevalence and determinants of pre-lacteal feeding in South Sudan: a community-based survey. Glob Health Action.

[21] Schmidt CO, Kohlmann T (2008). When to use the odds ratio or the relative risk?. Int J Public Health.

[22] Cameron AC, Trivedi PK (1990). Regression-based tests for overdispersion in the Poisson model. J Econ.

[23] Argaw MD, Asfaw MM, Ayalew MB, Desta BF, Mavundla TR, Gidebo KD, Frew AH, Mitiku AD, Desale AY (2019). Factors associated with prelacteal feeding practices in Debre Berhan district, north Shoa, Central Ethiopia: a cross-sectional, community-based study. BMC Nutrition.

[24] Kimani-Murage EW, Wekesah F, Wanjohi M, Kyobutungi C, Ezeh AC, Musoke RN, Norris SA, Madise NJ, Griffiths P (2015). Factors affecting actualisation of the WHO breastfeeding recommendations in urban poor settings in Kenya. Matern Child Nutr.

[25] Demisse TL, Aliyu SA, Kitila SB, Tafesse TT, Gelaw KA, Zerihun MS (2019). Utilization of preconception care and associated factors among reproductive age group women in Debre Birhan town, north Shewa, Ethiopia. Reprod Health.

[26] Abdel-Rahman ME, El-Heneidy A, Benova L, Oakley L (2020). Early feeding practices and associated factors in Sudan: a cross-sectional analysis from multiple Indicator cluster survey. Int Breastfeed J.

[27] Ogundele T, Ogundele OA, Adegoke AI (2019). Determinants of prelacteal feeding practices among mothers of children aged less than 24 months in Ile-Ife Southwest Nigeria: a community cross-sectional study. Pan Afr Med J.

[28] Amele EA, Wondimeneh Demissie B, Desta KW, Woldemariam EB (2019). Prelacteal feeding practice and its associated factors among mothers of children age less than 24 months old in Southern Ethiopia. Ital J Pediatr.

[29] Tessema GA, Mahmood MA, Gomersall JS, Assefa Y, Zemedu TG, Kifle M, Laurence CO (2020). Structural quality of services and use of family planning services in primary health care facilities in Ethiopia. How do public and private facilities compare?. Int J Environ Res Public Health.

[30] Republic of Uganda (2015). Health Sector Development Plan 2015/16–2019/2020.

[31] Republic of Uganda (2018). Annual health sector performance report: financial year 2017/2018.

[32] Geleto A, Chojenta C, Musa A, Loxton D (2018). Barriers to access and utilization of emergency obstetric care at health facilities in sub-Saharan Africa: a systematic review of literature. Syst Rev.

